# Efficacité de la prévention de la transmission mère-enfant du Virus de l'Immunodéficience Humaine par le protocole 2010 de l'Organisation Mondiale de la Santé au Centre Médical Saint Camille de Ouagadougou (Burkina Faso)

**DOI:** 10.11604/pamj.2015.22.303.7720

**Published:** 2015-11-25

**Authors:** Solange Odile OuédraogoYugbaré, Nikaise Zagré, Fla Koueta, Lassina Dao, Ludovic Kam, Diarra Yé Ouattara, Jacques Simporé

**Affiliations:** 1 Unité de Formation et de Recherche en Sciences de la Santé, Ouagadougou, Burkina Faso; 2 Centre Hospitalier Universitaire Pédiatrique Charles de Gaulle, Burkina Faso; 3 Service de pédiatrie du Centre Hospitalier Universitaire–Yalgado Ouédraogo; 4 Unité de Formation et de Recherche en Sciences de la vie et de la terre, Ouagadougou, Burkina Faso; 5 Centre médical saint Camille, Ouagadougou, Burkina Faso

**Keywords:** PTME, Option A, trithérapie, Burkina Faso, PMTCT, Option A, tritherapy, Burkina Faso

## Abstract

L’épidémie du Virus de l'Immunodéficience Humaine en milieu pédiatrique est surtout le résultat de la transmission mère-enfant. Notre étude a pour objectif de décrire l'efficacité de la prévention de la transmission mère-enfant du Virus de l'Immunodéficience Humaine par le protocole OMS 2010 (Option A et trithérapie) au centre médical saint Camille de Ouagadougou. Nous avons mené une étude d'une cohorte d'enfants suivis dans le cadre de la prévention de la transmission mère-enfant du Virus de l'Immunodéficience Humaine au centre médical saint Camille de Ouagadougou sur une période de 2 ans allant du 1er Janvier 2012 au 31 Décembre 2013. Nous avons obtenu l'accord de 4900 femmes enceintes pour le dépistage de l'infection du Virus de l'Immunodéficience Humaine et 238 gestantes ont été diagnostiquées séropositives soit 4,86% de séroprévalence. Les femmes étaient surtout infectées par le Virus de l'Immunodéficience Humaine avec de type I (95,38%)). La majorité était sous trithérapie (74,3%) et (25,7%) sous prophylaxie (option A). Les nouveau-nés (92,5%) ont reçu un traitement antirétroviral à base de névirapine dans les 72 heures après la naissance. L'allaitement sécurisé a été appliquée dans 78% des cas. Le taux global de transmission mère-enfant du VIH était de 3,6% avec 3% de transmission chez les enfants nés de mères sous trithérapie antirétrovirale et 6,3% dans les cas de prophylaxie antirétrovirale. Le facteur de risque de transmission a été le long délai du début d'administration des antirétroviraux chez le nouveau-né. La mortalité infantile à un an était de 3,5%. Cette étude a révélé l'efficacité de l'Option A et conforté celle de la trithérapie, le passage à l'Option B+ serait donc plus bénéfique.

## Introduction

Le syndrome d'immunodéficience acquise (SIDA) est une infection par le Virus de l'Immunodéficience Humaine (VIH). Elle est responsable d'un déficit immunitaire et de manifestations cliniques variables. A l’échelle mondiale, on estimait à 34,0 millions le nombre de personnes vivant avec le VIH/SIDA en fin 2011 dont 3,4 millions enfants de moins de 15 ans [1]. L'Afrique Subsaharienne était la plus touchée avec 23,5 millions soit 69% des personnes infectées dans le monde. On y dénombrait 200000 nouvelles infections soit en moyenne 600 enfants infectés par jour qui représentaient 90% des transmissions du VIH chez les enfants au plan mondial [2]. L’épidémie pédiatrique à VIH est surtout le résultat de la transmission mère-enfant du VIH. Au Burkina Faso, des stratégies nationales ont été élaborées dépuis 2000 incluant un programme de PTME qui a connu des perfections au fil du temps selon les différentes recommandations et normes de l'organisation mondiale de la santé (OMS). Ainsi trois protocoles de prévention de transmission mère enfant (PTME) ont été appliqué jusqu’à ce jour. Le protocole en vigueur depuis 2012 est celui de l'OMS 2010 (Option A) [3]. Le Centre Médical Saint Camille (CMSC) qui est un des centres pilote au Burkina Faso de référence dans la PTME du VIH/SIDA avait un taux de 10,4% en 2006. Nous nous proposons à travers la présente étude de décrire l'efficacité dans la PTME du protocole OMS 2010 (Option A) du VIH/SIDA dans cette structure.

## Méthodes

Il s'est agi d'une étude rétrospective d'une cohorte de gestantes du 01er Janvier 2012 au 31 Décembre 2013 au Centre Médical Saint Camille et au laboratoire du Centre de Recherche Biomoléculaire Pietro Annigoni (CERBA). Ont été incluses dans l’étude par échantillonnage aléatoire simple toutes les gestantes séropositives au VIH (nouvellement dépistées à l'issu du Counseling Volontaire ou antérieurement suivies au CMSC) qui se sont présentées à la SMI pendant la période d’étude, les gestantes infectées, référées pour le suivi de la grossesse au CMSC par d'autres centres de prise en charge de PvVIH. La sérologie a été déterminée après le Conseil Dépistage Volontaire par un test rapide Determine (Determine HIV-1/2 test, Alère GmbH, Koln, United, Kingdom) et SD-Bioline (SD-Bioline HIV-1/2 antibody test^TM^ 3.0, Standard Diagnostics, Inc.). Les gestantes ayant un résultat négatif ont été invités à reprendre le test trois mois plus tard. Pour les résultats indéterminés la polymérisation chain réactive (PCR) a été demandée. Les femmes enceintes séropositives au VIH nouvellement dépistées ont été divisé en 2 groupes qui ont reçus une trithérapie ou une prophylaxie antirétrovirale (ARV) en fonction de leurs états clinique et biologique. La prophylaxie a consisté à l'administration de la zidovidune (AZT) 300mg matin et soir à partir de la 14^ème^ semaine d'amenorrhée (SA) et pendant le travail de la névirapine(NVP) 200mg en prise unique et de la zidovidune /lamivudine (AZT/3TC) 300mg/150mg (DUOVIR^®^). Le DUOVIR^®^ a été renouvelé deux fois par jour pendant la première semaine du post-partum. La trithérapie zidovidune /lamivudine/névirapine (AZT/3TC/NVP) ou zidovidune /lamivudine/éfavirenz (AZT/3TC/EFN) était instituée très tôt quelque soit l’âge gestationnel puis maintenue tout au long de la grossesse, de l'accouchement et en post natal. Tous les nouveau- nés au CMSC ont été hospitalisés en néonatologie les 72 premières heures de vie puis suivis régulièrement, deux fois le premier mois puis une fois par mois durant la première année. Ils ont reçu quotidiennement de la NVP (4mg/kg/jour en prise unique) de la naissance à une semaine après l'arrêt de l'allaitement maternel pour ceux nourris au lait maternel et de la naissance à 6 semaines de vie pour ceux alimentés aux substituts du lait maternel. Au cours du suivi, une PCR a été réalisée entre deux et six mois et la sérologie au 12^ème^ et au 18^ème^ mois. En cas d’échec de la PTME (PCR ou sérologie positive), l'enfant était suivi par la section pédiatrique de la prise en charge des personnes vivant avec le VIH (PvVIH) au CMSC. Les femmes ont été prises en charge dans le respect strict de la confidentialité et leur consentement a été obtenu pour le dépistage. Le test de Chi-carré et de Fischer ont été réalisé pour comparer les variables catégorielles avec un seuil de significativité de 5%.

## Résultats

**Figure 1:**
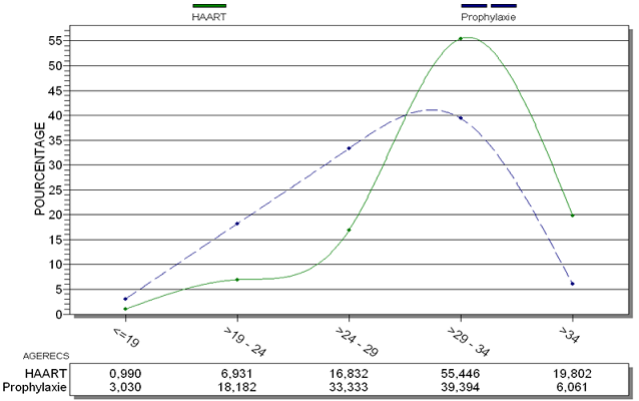
Répartition des femmes en fonction de l’âge et du traitement

Durant les 24 mois de l’étude, 4900 gestantes reçues à la SMI, ont accepté de faire le dépistage du VIH, 238/4900 ont été testées séropositives soit une séroprévalence de 4,86%. Parmi ces gestantes, 19,75% (47/238) femmes découvraient pour la première fois leur statut sérologique. Le VIH-1 était prédominant (95,38%) suivi du VIH-2 (2,94%) et du VIH1&2 (1,68%) (Tableau 1). L’âge moyen des mères était de 30,93 ans avec des extrêmes allant de 17 à 42 ans. Il y'avait une différence statistiquement significative de moyenne d’âge entre les deux options thérapeutiques avec 31,90 ans pour la trithérapie et 28,14 ans pour la prophylaxie (p=0,0004). La classe d’âge de ]29-35] était prédominante dans les deux options de traitement (Figure 1). Les mères étaient surtout des ménagères (60,3%) avec 65,3% de ménagères pour la trithérapie et 45,7% pour la prophylaxie. La moyenne de parité était de 3 accouchements chez les femmes sous trithérapie et de 2 accouchements chez les femmes sous option A. Il existait une différence statistiquement significative entre ces deux groupes de femme (p<0,00001). Il y’ a 136 gestantes qui ont poursuivies leur consultation prénatale au CMSC.Nous avons pu classer 81/136 femmes selon les stades cliniques de l'OMS. Toutes les femmes sous option A ont été classées stade clinique I de l'OMS, et les femmes sous trithérapie à 81,5% au stade clinique I et II de l'OMS. Le taux de CD4 variait de 65 à 1670 cellules/µl avec un taux moyen de 502,49 cellules/µl (±245,34). La moyenne de CD4 sous trithérapie était de 500,19 cellules/µl (±258,30) et de 498,33 cellules/µl (±218,74) en prophylaxie mais il n'y avait pas de différence statistiquement significative ( P>0,05). La date de mise des ARV a été renseignée chez 28/35 femmes sous prophylaxie et 46,4% ont reçu leurs ARV aux environs de la 14^ème^ semaine d'aménorrhée (entre 3 et 4 mois) conformément au protocole. Parmi les 136 femmes, 114 naissances vivantes ont été enregistrées, soit 85/136 en trithérapie et 29/136 en prophylaxie. L'accouchement par césarienne a été noté chez 2,8% (3/108) des femmes. Toutes les femmes sous prophylaxie (28/28) ont accouché par voie basse. La prématurité a été notée chez 7,89% (9/114) naissances vivantes repartie en 8,24% mères sous trithérapie soit et 6,90%. Sous prophylaxie. Le faible poids de naissance retrouvé dans 16,10% (9/56) cas a été enregistré chez 16,66% à des enfants nés de mères sous trithérapie et 14,29% de ceux de mères sous prophylaxie mais la différence n’était pas significative (p>0,82). La majorité des femmes ont fait un choix éclairé de l'allaitement sécurisé pour leurs enfants soit 78,95% (90/114). Tous les enfants qui ont reçu des ARV (111/114) dès la naissance ont été mis uniquement sous Névirapine sirop selon le protocole. En fonction du délai d'administration de la NVP, 92,79% (103/111) des nouveau-nés ont reçu leur première dose dans les 24 à 48H et 7,21% (8/111) des enfants l'ont reçu plus d'une semaine après. En fonction du lieu d'accouchement (centre spécialisé dans la PTME/VIH ou non), il existait une différence statistiquement significative du délai d'administration de la NVP (p<0,001). Notre étude a noté un taux de transmission verticale global de 3,61% (3/83) avec 5,88% de nouveau- nés de mères sous trithérapie et 3,3% de nouveau- nés de mères sous prophylaxie. Le taux de transmission du VIH était 3,08% chez les enfants nourris au sein et de 5,56% en cas d'alimentation (p>0,05). Le taux de mortalité était de 3,5% avec 2,67% dans les 6 premières semaines. Les données de l'accouchement et du nouveau ‘né à la naissance sont illustrés par le Tableau 2 sur les 109 enfants vivant à 6 semaines 76,85% (83) enfants ont bénéficié d'un dépistage du VIH par PCR entre 6 semaines et 4 mois. Ces PCR étaient positives chez 3,61% (3/83) enfants répartie en 3,03% (2/66) pour la trithérapie et 5,88% (1/17) pour la prophylaxie mais la différence n’était pas statistiquement significative (p=0,50). A 12 mois et 18 mois 17 enfants ont bénéficié d'un test de dépistage rapide du VIH qui était tous négatifs. Tous les enfants ayant bénéficiés de test rapide, avaient auparavant réalisés une PCR était négative. Le statut virologique de l'enfant n’était pas en corrélation avec: le mode d'accouchement, lieu d'accouchement, terme de l'enfant, le poids de naissance, la réalisation d’épisiotomie, l’état des membranes des eaux, le mode d'alimentation. Seul le délai de la prise des ARV à la naissance était statistiquement lié à la transmission mère-enfant du VIH avec p=0,0051(Tableau 3).

**Tableau 1:** Fréquence du VIH et du type de VIH en fonction de l’âge des mères

AGE	Gestantes	VIH NEG		VIH POSITIF					
				VIH 1		VIH 2		VIH 1&2	
	Effectif	Effectif	%	Effectif	%	Effectif	%	Effectif	%
**≤ 19**	**296**	292	98,65	4	1,35	0	0,00	0	0,00
[20-24]	**1596**	1564	97,99	32	2,01	0	0,00	0	0,00
[25-29]	**1729**	1657	95,84	68	3,93	1	0,06	3	1,17
[30-35]	**896**	791	88,28	9	11,05	5	0,56	1	0,11
>35	**383**	358	93,47	24	6,27	1	0,26	0	0,00
**Total**	**4900**	**4662**	**95,14**	**227**	**4,6**	**7**	**0,14**	**4**	**0,08**

**Tableau 2:** Données de l'accouchement et du nouveau –né à la naissance

	HAART		Prophylaxie		Total	
	effectif	pourcentage	effectif	pourcentage	effectif	pourcentage
**Prématurité**	7/85	8,24%	2/29	6,90%	9/114	7,89%
**Faible Poids**	7/42	16,66%	2/14	14,29%	9/56	16,10%
**Episiotomie**	15/42	35,71%	6/14	42,86%	21/56	37,5%
**Rupture artificielle**	12/42	28,57%	2/12	16,67%	14/54	25,93%
**LA méconial**	12/42	28,57%	5/13	38,46%	17/55	30,91%
**Allaitement**	66/85	77,65%	24/29	82,76%	90/114	78,95%
**Décès à 6 semaines**	1/83	1,20%)	2/29	6,90%	3/112	2,67%

**Tableau 3:** Facteurs associés à la transmission mère enfant de l'infection à VIH

FACTEURS	TME		P
	effectif	pourcentage	
AGE			
] 19-24]	1/7	14,29	
[25-29]	1/20	5	
[30-34]	1/36	2,78	
≥35	0/17	0	0,39(NS)
PROFESSION			
Ménagère	2/49	4,08	
Commerçante	0/14	0	
Etudiante/Elève	0/2	0	
Fonctionnaire	0/11	0	
Autres	1/3	3,33	0,19(NS)
PARITE			
Primipare	0/8	0	
Paucipare	2/46	4,35	
Multipare	1/22	4,55	
Grande multipare	0/2	0	0,93(NS)
MODE D'ACCOUCHEMENT			
Voie basse	3/78	3,85	
Césarienne	0/2	0	0,93(NS)
MATURITE			
Prématuré	0/4	0	
A Terme	3/79	3,80	0,86(NS)
ASPECT DU LA			
Clair	1/25	4	
Sanglant	0/4	0	
Méconial	0/12	0	
Autres	0/1	0	0,95(NS)
ETAT DES MEMBRANES			
Rupture avant entrée	0/4	0	
Rupture spontanée	1/24	4,17	
Rupture artificielle	0/12	0	0,85(NS)
POIDS			
<2500g	0/8	0	
2500-4000g	1/36	2,78	0,82(NS)
ALIMENTATION			
Maternel sécurisé	2/65	3,08	
Artificiel	1/18	5,56	0,53(NS)
Début ARV chez le nouveau-né			
24-48H	1/79	1,27	
> 7 Jours	2/4	0,5	***0,0051***

## Discussion

Notre prévalence de 4,86% est inférieure à celui d'Ilboudo (Burkina Faso 2010) [4] qui a retrouvé une prévalence de 5,40% au CMSC identique à la prévalence nationale (5,40% en 2012) [3]. Cependant cette baisse relative s'explique par la forte fréquentation du CMSC qui est à but social; aussi parce qu'il s'agit d'un centre pilote nationale de la PTME, reconnu pour la qualité des soins qui y sont dispensés. Ainsi KIRSTEN et al, (Tanzani 2011) [5] dans un centre similaire au CMSC, enregistrait une prévalence de 14,50% alors que la prévalence nationale était de 6,00%. L’âge moyen des femmes était de 30,93 ans. Nos résultats sont comparables à ceux de Tougri (Burkina Faso 2008) [6]; Kouam et al (Cameroun 2006) [7] qui notaient 29 ans et 27,70 ans. Le VIH est une affection du sujet jeune, sexuellement actif avec des comportements à risque. Les mères étaient surtout des femmes au foyer c (60,3%) comme pour Semporé (Burkina Faso 2008) (70,0%) [8], et Koanda et al., (Burkina Faso 2010) (75,5%) [9]. Ceci est le reflet des conditions de vie de la femme dans notre pays à cause de la sous scolarisation des filles, l'inégalité entre homme et femme dans la recherche de l'emploi et le refus de certains hommes de laisser leurs femmes travailler hors du foyer.

Toutes les femmes sous option A ont été classées au stade clinique I de l'OMS et 81,5% de celles sous trithérapie se répartissaient majoritairement entre le stade I et II. Semporé (Burkina Faso 2008) [8] avait rapporté 83,2% pour les stades II. Les stades III et IV seraient peu compatibles avec la fertilité Le taux moyen de CD4 était à 502,49 cellules/µl, révélant un bon état immunitaire des gestantes. Cette moyenne est comparable à celles de Tonwe-Gold (République Côte d'Ivoire 2007) (467 cells/µl) [10] de Semporé(Burkina Faso 2008) (446,25 cells/µl) [8]. Une question d’éthique pourrait donc se poser au vu du bon état immunitaire des gestantes, serait-ce acceptable d'exposer celles-ci et de façon définitive aux effets secondaires de la trithérapie’ La majorité des femmes sous trithérapie (49,4%) ont fait le dépistage au premier trimestre de la grossesse. Ouoba (Burkina Faso 2007) [11] et Semporé (Burkina Faso 2008) [8] avaient trouvé respectivement 50,0% et 48,5% de dépistage au deuxième trimestre à Ouagadougou et au CMSC. Ce fort taux de dépistage précoce est dû au fait que la majorité des femmes connaissait leur statut sérologique et était suivie dans des centre de prise en charge des PvVIH. Elles savaient donc les risques liés à leur état et la nécessité d'un suivi précoce de la grossesse. Par contre les femmes sous prophylaxie ignoraient pour la plupart leur statut sérologique VIH et se présentaient en consultation qu'après apparition de signes évidents de grossesse. Ceci expliquerait que le début du traitement ARV à 14^ème^ SA n'a été effective que chez 46,7% des femmes mises sous prophylaxie. Semporé(Burkina Faso 2008) [8] avait retrouvé 54,8% de femmes qui avaient reçu les ARV dans les délais requis (28^ème^ SA) et expliquait les retards par: l'ignorance de l’âge gestationnel (rareté de l’échographie obstétricale) et le non partage des résultats de la sérologie VIH avec le conjoint par peur de rejet et de stigmatisation. Le lieu d'accouchement était corrélé de façon significative avec le délai de prise de la première dose de Nevirapine du nouveau-né (p=0,0001). Cela traduit le manque de connaissance du statut sérologique de la mère par le personnel reflétant une défaillance du système de communication inter-service et une insuffisance dans l'interrogatoire. Aussi il y'a la peur de la découverte de la sérologie de la mère par les accompagnants Dans notre étude la majorité des femmes (78,95%) ont opté pour l'allaitement sécurisé: Abordable, Faisable, Acceptable, Durable et Sûr(AFADS) pour leurs enfants. Nos résultats sont opposés à ceux de Semporé (Burkina Faso 2008) [8] et de Tougri (Burkina Faso 2008) [6] qui notaient respectivement 91,4% et 75,7% pour l'alimentation artificielle. La promotion de l'allaitement maternel exclusif s'impose dans notre contexte socioculturel à cause de ses multiples avantages. Le traitement ARV (NVP sirop) a été administré chez tous les enfants avec parfois des retards de plus d'une semaine. La principale cause de ce retard est l'accouchement dans un site non spécialisé dans la PTME (p<0,001). Trois enfants n'ont pas reçu de névirapine à la naissance posant ainsi le problème de l'insuffisance de communication entre les agents de santé et la peur de la stigmatisation par les mères. Notre étude a noté un taux de transmission verticale global de 3,61% (383). Ce taux est inférieur à celui du niveau national qui était de 5,96% en 2012 [3]. Le seul facteur de risque statistiquement lié à la morbidité était le long délai dans l'administration de la NVP chez les nouveau-nés (p= 0,0051). Si le protocole avait été bien respecté (première dose de NVP dans les 6 à 12H) nous aurions pu éviter la TME chez ces enfants. Le taux de transmission du VIH était 3,08% chez les enfants nourris au sein et de 5,56% en cas d'alimentation (p>0,05). Nos résultats sont contraires à ceux de Kouanda en 2010 [9]. Ainsi ce protocole valorise l'allaitement qui AFADS dans notre contexte est très bénéfique pour l'enfant. Le taux de mortalité était surtout précoce (2,67%). Aucun facteur de risque (prématurité, souffrance fœtale aigue, faible poids de naissance) n’était statistiquement associé à la mortalité Albrecht (Zambie 2006) [12] enregistrait un taux de 5,8% au cours du premier mois, ceci est le reflet de la mortalité néonatale des pays en voie de développement.

## Conclusion

Le protocole OMS de la PTME dans son Option A a permis de réduire d'une manière significative la transmission verticale du VIH et de préserver l'allaitement au sein qui est convenable pour notre société. L'Option B+ en vigueur depuis août 2014 au Burkina Faso permettra de maintenir plus longtemps les mères sous traitement et d'augmenter leur survie et celle de leurs enfants. Ainsi l'objectif des « trois zéro » voulu par l'OMS est donc possible: zéro mort, zéro contamination, zéro stigmatisation d'ici 2015.
